# Fasting-induced G0/G1 switch gene 2 and FGF21 expression in the liver are under regulation of adipose tissue derived fatty acids

**DOI:** 10.1016/j.jhep.2015.02.035

**Published:** 2015-08

**Authors:** Doris Jaeger, Gabriele Schoiswohl, Peter Hofer, Renate Schreiber, Martina Schweiger, Thomas O. Eichmann, Nina M. Pollak, Nadja Poecher, Gernot F. Grabner, Kathrin A. Zierler, Sandra Eder, Dagmar Kolb, Franz P.W. Radner, Karina Preiss-Landl, Achim Lass, Rudolf Zechner, Erin E. Kershaw, Guenter Haemmerle

**Affiliations:** 1 Institute of Molecular Biosciences, University of Graz, Heinrichstrasse 31, A-8010 Graz, Austria; 2 Division of Endocrinology, Diabetes, and Metabolism, University of Pittsburgh, PA 15261, USA; 3 ZMF, Center for Medical Research, Medical University of Graz, A-8010 Graz, Austria

**Keywords:** AT, Adipose tissue, FA(s), fatty acid(s), TG, triacylglycerol, CGI-58, comparative gene identification-58, ATGL, Adipose triglyceride lipase, *CGI-58-*ATko, AT-selective ablation of CGI-58, *ATGL-*ATko, AT-specific deficiency of ATGL, LD, lipid droplet, NAFLD, nonalcoholic fatty liver disease, NLSD, Neutral lipid storage disease, PPARα, peroxisome proliferator-activated receptor alpha, CREBH, cAMP-responsive element binding protein, hepatocyte specific, G0S2, G0/G1 Switch Gene 2, FGF21, fibroblast growth factor 21, PGC-1α, PPARgamma co-activator-1alpha, HNF4α, hepatocyte nuclear factor 4alpha, PEPCK, Phosphoenolpyruvate carboxykinase, G6Pase, Glucose-6-phosphatase, ER, endoplasmic reticulum, Hepatic steatosis, G0/G1 switch gene 2, Fibroblast growth factor 21, CGI-58, ATGL, Lipolysis, PPARα, CREBH, Obesity

## Abstract

**Background & Aims:**

Adipose tissue (AT)-derived fatty acids (FAs) are utilized for hepatic triacylglycerol (TG) generation upon fasting. However, their potential impact as signaling molecules is not established. Herein we examined the role of exogenous AT-derived FAs in the regulation of hepatic gene expression by investigating mice with a defect in AT-derived FA supply to the liver.

**Methods:**

Plasma FA levels, tissue TG hydrolytic activities and lipid content were determined in mice lacking the lipase co-activator comparative gene identification-58 (CGI-58) selectively in AT (*CGI-58-*ATko) applying standard protocols. Hepatic expression of lipases, FA oxidative genes, transcription factors, ER stress markers, hormones and cytokines were determined by qRT-PCR, Western blotting and ELISA.

**Results:**

Impaired AT-derived FA supply upon fasting of *CGI-58-*ATko mice causes a marked defect in liver PPARα-signaling and nuclear CREBH translocation. This severely reduced the expression of respective target genes such as the ATGL inhibitor G0/G1 switch gene-2 (G0S2) and the endocrine metabolic regulator FGF21. These changes could be reversed by lipid administration and raising plasma FA levels. Impaired AT-lipolysis failed to induce hepatic G0S2 expression in fasted *CGI-58-*ATko mice leading to enhanced ATGL-mediated TG-breakdown strongly reducing hepatic TG deposition. On high fat diet, impaired AT-lipolysis counteracts hepatic TG accumulation and liver stress linked to improved systemic insulin sensitivity.

**Conclusions:**

AT-derived FAs are a critical regulator of hepatic fasting gene expression required for the induction of G0S2-expression in the liver to control hepatic TG-breakdown. Interfering with AT-lipolysis or hepatic G0S2 expression represents an effective strategy for the treatment of hepatic steatosis.

## Graphical abstract

**Figure:**
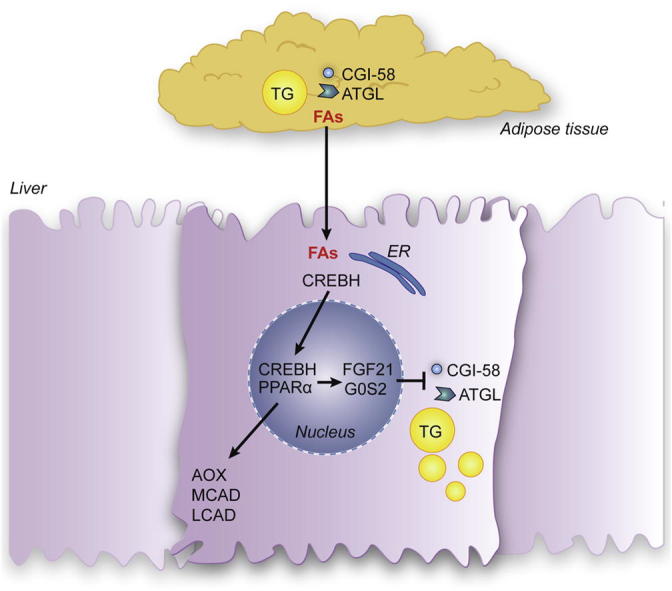

## Introduction

In mammals, the deposition and mobilization of triacylglycerol (TG) from adipose tissue (AT) plays a central role in energy homeostasis. This steadily recurring metabolic circuit is sensed by the liver and reflected by continuous changes in hepatic TG content and gene expression. Imbalances in this process may lead to pronounced fat accumulation in the liver eventually progressing to non-alcoholic fatty liver disease (NAFLD).

The catabolism of endogenous TG deposited within cellular lipid droplets (LDs) depends on adipose triglyceride lipase (ATGL) [1] and a co-activator designated as comparative gene identification-58 (CGI-58) [2]. Both, mutated *ATGL* or *CGI-58* alleles promote neutral lipid storage disease (NLSD) characterized by TG accumulation in multiple tissues [3] including the liver. In mice, downregulation or absence of ATGL or CGI-58 in the liver provokes marked hepatic fat accumulation [4], [5], [6]. Upon fasting, hepatic TG levels sharply increase due to enhanced TG mobilization from AT and increased fatty acid (FA) flow to the liver. Excess hepatic TG in NAFLD patients substantially originates from AT-derived FAs [7]. However, the functional importance of AT-derived FAs in the regulation of hepatic gene expression is still unclear and challenged by the study of Chakravarthy *et al.* [8] suggesting that PPARα-regulated gene expression in the liver depends on *de novo* FA synthesis. The aim of this study was to unravel the *in vivo* role of exogenously supplied FAs in the regulation of hepatic gene expression and TG homeostasis upon fasting.

Here we show that AT-derived FA supply is critically required to stimulate expression of genes in the liver which are under the dual regulation of PPARα and cAMP-responsive element binding protein H (CREBH) encompassing the lipase inhibitor G0S2 and the endocrine metabolic regulator fibroblast growth factor 21 (FGF21) which has major implications on hepatic TG catabolism and systemic energy homeostasis.

## Materials and methods

### Mouse models

*CGI-58*-floxed mice [9] and *Fabp4*-Cre transgenic mice [10] have previously been described and used for the generation of mice with AT-selective disruption of CGI-58 (*CGI-58*-ATko). Mice lacking ATGL specifically in AT (*ATGL-*ATko) were generated by breeding the *AdipoQ-Cre* transgene onto an *ATGL*-floxed background [11]. Maintenance of mice is described in detail in the Supplementary Material.

### Analysis of body composition

Body composition of non-fasted animals was determined using a calibrated Bruker miniSpec NMR analyzer (Bruker Optics).

### Blood parameters

Determination of plasma parameters is described in the Supplementary Material.

### PPARα-agonist application and raising plasma FA levels

The established PPARα agonist Wy14643 was injected (50 μg/g body weight) into 12 h fasted mice. After another 2 h fasting the liver was surgically removed for RNA isolation and qRT-PCR. For raising circulating FA levels, an intragastric olive oil gavage (200 μl/mouse) was administered to 12 h fasted mice followed by heparin injection (10 IU/mouse). After another 2 h, blood was collected and liver tissues were harvested.

### Analysis of gene expression by qRT-PCR

qRT-PCR and Xbp1-splicing assay was performed as described in the Supplementary Material.

### Measurement of tissue TG levels

Tissue lipids were extracted with chloroform and methanol and determined as described in the Supplementary Material.

### Western blotting

Western blot analyses were performed according to standard protocols described in the Supplementary Material.

### Electron microscopy

Electron microscopy has been performed as previously described [12].

### Statistical analysis

Statistical significance was determined by the Student’s unpaired *t* test (two-tailed). Group differences considered significant for *p <*0.05 (^∗^), *p <*0.01 (^∗∗^), and *p <*0.001 (^∗∗∗^).

## Results

### AT-derived FAs are critically required to induce G0S2 expression in the liver to regulate hepatic TG catabolism upon fasting

**Fig. 1:**
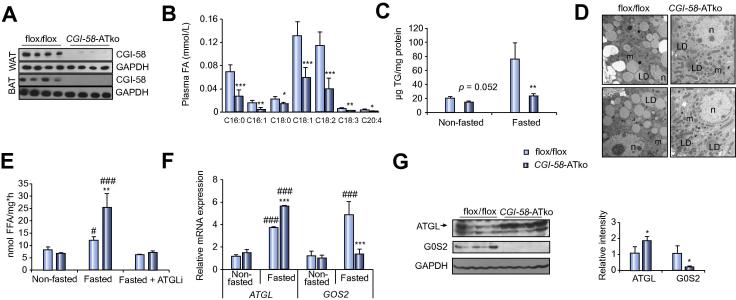
**AT-derived FAs induce G0S2 expression in the liver to regulate hepatic TG catabolism.** (A) Absence of CGI-58 protein expression in white (gonadal) and brown AT (WAT and BAT) of *CGI-58-*ATko mice. (B) Plasma concentrations of saturated and unsaturated FA species in fasted flox/flox and *CGI-58-*ATko mice. (C) Hepatic TG levels in non-fasted compared to fasted flox/flox and *CGI-58-*ATko mice. (D) Transmission electron microscopy of liver sections from fasted mice (Scale bar = 1 μm; LD, lipid droplet; m, mitochondria; n, nucleus). (E) TG hydrolytic activities in liver preparations of non-fasted compared to fasted mice and in the presence of an ATGL-specific inhibitor (ATGLi). (F) Hepatic *ATGL* and *G0S2* mRNA expression in non-fasted compared to fasted mice. (G) Hepatic ATGL and G0S2 protein expression upon fasting. Values are mean ± SD from at least 5 mice per genotype. ^*^p <0.05, ^**^p <0.01, and ^***^p <0.001 *vs.* flox/flox; ^*#*^*p *<0.05 and ^*###*^*p *<0.001 non-fasted *vs.* fasted.

Efficient cellular TG catabolism depends on ATGL and the lipolytic co-activator CGI-58 [1]. To restrict FA release from AT, we generated mice lacking CGI-58 selectively in AT (*CGI-58-*ATko) by breeding *Fabp4*-Cre transgenic mice [10] with mice carrying *loxP*-sites in the *Cgi-58* gene [9]. CGI-58 expression was substantially reduced in gonadal white and brown AT of *CGI-58-*ATko mice (Fig. 1A; Supplementary Fig. 1A) whereas muscle and hepatic CGI-58 mRNA levels were comparable to flox/flox controls. Body weight of *CGI-58-*ATko mice was similar to flox/flox controls (Supplementary Fig. 1B) whereas body fat mass was 1.4-fold increased paralleled by elevated gonadal and brown AT and reduced liver mass (Supplementary Fig. 1C and D). Fasting provoked a marked decrease in plasma FA (−66%), TG (−58%) and glycerol levels in *CGI-58-*ATko mice (Table 1). Plasma levels of long-chain saturated and unsaturated FA species were significantly decreased (ranging from −29% up to −73%) (Fig. 1B). The reduction in blood glucose and plasma insulin levels together with lower HOMA-IR indexes in *CGI-58-*ATko compared to flox/flox mice (Table 1) indicates increased insulin sensitivity. Hepatic glycogen content was markedly reduced (−74%) in 6 h fasted *CGI-58-*ATko compared to flox/flox mice (3.6 ± 5.6 *vs.* 13.5 ± 6.1 mg glucose per g tissue, respectively). Together, these data validate *CGI-58-*ATko mice to study the impact of defective AT-lipolysis on hepatic TG homeostasis and gene expression.

**Table 1:** **Plasma energy metabolites in non-fasted compared to fasted flox/flox and *CGI-58-*ATko mice on chow. Plasma glucose, insulin and HOMA-IR refer to 6 h fasted mice.**

Values represent mean ± SD; ^*^p <0.05, ^**^p <0.01, ^***^p <0.001 (n ⩾6).

Hepatic TG (Fig. 1C) and total cholesterol (Supplementary Fig. 1E) levels were unchanged in non-fasted *CGI-58-*ATko compared to flox/flox mice whereas TG levels were strongly reduced (−71%) in fasted *CGI-58-*ATko mice compared to controls. Examination of liver tissue morphology by transmission electron microscopy (Fig. 1D) revealed very low abundance of LDs in liver sections of fasted *CGI-58-*ATko mice and counting of LDs showed a 9.2-fold reduction in LD numbers of nearly all sizes (Supplementary Fig. 1F). Notably, TG hydrolytic activities (Fig. 1E) were exclusively and markedly increased in liver preparations of fasted *CGI-58-*ATko compared to control mice, and addition of a specific ATGL inhibitor [13] to tissue preparations reduced activities to flox/flox levels suggesting increased ATGL-mediated TG-breakdown. In accordance with this assumption, hepatic ATGL expression was markedly increased in fasted *CGI-58-*ATko mice (up to 1.7-fold) whereas expression of the ATGL inhibitor G0S2 [14] was drastically reduced on the mRNA (−3.7-fold) and protein level (−5.5-fold) (Fig. 1F and G). The strong reduction of hepatic G0S2 expression suggests that the increase in hepatic TG breakdown is mainly due to failed induction of hepatic G0S2 expression in the liver. To corroborate this conclusion and to confirm that CGI-58 disruption affected hepatic gene expression via defective AT-lipolysis, we measured G0S2 protein levels in *ATGL-*ATko mice which significantly impairs AT-lipolysis in the fasted state [15], [16]. ATGL was disrupted in AT by breeding *Atgl*-floxed mice [11] with *AdipoQ*-*Cre* transgenic mice. In line with *CGI-58-*ATko mice, hepatic G0S2 expression was strongly reduced whereas ATGL expression was increased in fasted *ATGL-*ATko mice (Supplementary Fig. 1G).

### Fasting-induced expression of PPARα-regulated genes in the liver including FGF21 depends on peripheral FA supply

**Fig. 2:**
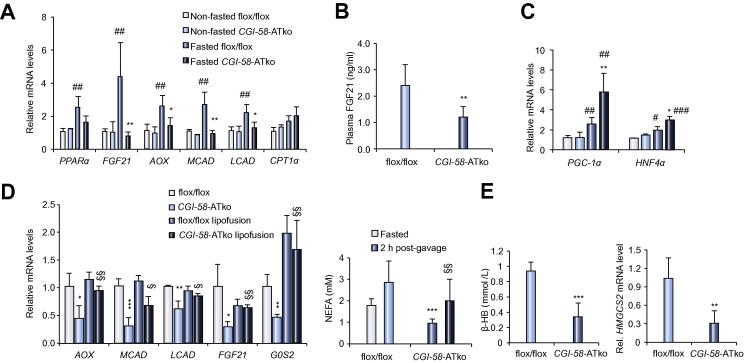
**Hepatic FGF21 and PPARα-regulated gene expression depends on exogenous FA supply.** (A) Hepatic mRNA expression of PPARα-regulated genes in non-fasted compared to fasted mice determined by qRT-PCR. (B) Plasma FGF21 concentrations in fasted flox/flox and *CGI-58-*ATko mice. (C) Hepatic *PPARα* and *HNF4α* mRNA expression determined by qRT-PCR. (D) mRNA levels of PPARα-regulated genes in the liver of fasted mice following intragastric olive oil administration and heparin injection which raises plasma FA levels (right panel). (E) Fasting plasma levels of β-hydroxybutyrate (β-HB) and (F) hepatic *HMGCS2* mRNA expression. Values are mean ± SD from at least 5 mice per genotype.^*^p <0.05, ^**^p <0.01, and ^***^p <0.001 *vs.* flox/flox; ^*#*^*p *<0.05, ^*##*^*p *<0.01, and ^*###*^*p *<0.001 non-fasted *vs.* fasted; ^§^*p *<0.05 and ^§§^*p *<0.01 *CGI-58-*ATko fasted *vs.* lipid and heparin administration in *CGI-58-*ATko mice.

The defect in AT-lipolysis profoundly interfered with hepatic TG catabolism via lack of fasting-induced G0S2 expression in the liver of *CGI-58-*ATko mice. Hepatic G0S2 mRNA expression is under the regulation of PPARα [17] indicating that AT-derived FAs impact PPARα-regulated gene expression in the liver. As shown in Fig. 2A, hepatic mRNA levels of *PPARα* and genes regulated or co-regulated by PPARα including *FGF21*, acyl-CoA oxidase (*AOX1*), medium-chain acyl-CoA dehydrogenase (*MCAD*) and long-chain acyl-CoA dehydrogenase (*LCAD*) were significantly decreased in the liver of overnight-fasted *CGI-58-*ATko mice (ranging from −35% to −86%) compared to levels of flox/flox mice. In contrast, mRNA expression of PPARα-regulated genes was similar in non-fasted *CGI-58-*ATko compared to control mice. In line with low mRNA expression, plasma FGF21 concentration was reduced by 48% (Fig. 2B). Next we examined expression of the transcriptional co-activator *PGC-1α* and hepatocyte nuclear factor 4alpha (*HNF4α*) which are highly expressed in the liver and bind to FAs [18]. Interestingly, and in contrast to reduced PPARα target gene expression, hepatic *PGC-1α* and *HNF4α* expression increased in fasted *CGI-58-*ATko compared to flox/flox mice on the mRNA (Fig. 2C) and nuclear protein level (Supplementary Fig. 2A and B). Unlike impaired PPARα-target gene expression in the liver, defective AT-lipolysis in *CGI-58-*ATko mice does not interfere with PPARα-regulated gene expression including FGF21 in cardiac and skeletal muscle (Supplementary Fig. 2C and D), which is in accordance with results of previous studies showing that PPARα-activation in cardiac muscle is not affected by low levels of exogenous FAs [9], [12]. As “proof of principle” we examined PPARα-regulated gene expression in the liver of fasted *ATGL-*ATko mice which also exhibit a marked defect in AT-lipolysis. AT-specific ATGL disruption showed an essentially identical hepatic mRNA expression profile except that *PGC-1α* mRNA expression was even more strongly increased (8.1-fold) compared to flox/flox controls (Supplementary Fig. 2E and F) further corroborating the critical role of AT-lipolysis in the regulation of hepatic fasting gene expression. To examine whether these changes in hepatic gene expression are directly mediated by low exogenous FA levels we followed a strategy where we increased circulating FA levels in fasted mice via intragastric olive oil administration followed by heparin injection to release lipoprotein lipase (LPL) thereby increasing TG-breakdown and circulating FA levels. Notably, 2 h after the lipid administration, hepatic mRNA expression of selected PPARα-regulated genes including *FGF21* significantly increased in *CGI-58-*ATko mice (Fig. 2D) and the effect was most pronounced for *G0S2*. These changes were paralleled by increased FA levels in *CGI-58-*ATko (and flox/flox) mice implicating that the increase in PPARα target mRNA expression was linked to the raise in circulating FA levels.

Reduced circulating FGF21 levels and the increase in hepatic PGC-1α and HNF4α expression in *CGI-58-*ATko mice prompted us to further examine the consequences on energy metabolism. FGF21 induces ketogenic gene expression in the liver and plays a critical role in regulating body temperature [19]. Impaired hepatic FGF21 expression in *CGI-58-*ATko mice was paralleled by significantly reduced (−66%) plasma ketone body concentrations (β-hydroxybutyrate) (Fig. 2E) and reduced (−69%) mRNA expression of *HMG-CoA synthase-2* (Fig. 2F), the rate-limiting enzyme in ketogenesis. Next we tested the impact of fasting on cold adaptation of *CGI-58-*ATko mice (Supplementary Fig. 3A and B). In contrast to the non-fasted state, fasting provoked a rapid decline in body temperature of *CGI-58-*ATko mice which disposed us to terminate the experiment. Hepatic gluconeogenesis is under the dual regulation of PGC-1α and HNF4α [20]. mRNA expression of *PEPCK* and *G6P* (Supplementary Fig. 3C) was increased in *CGI-58-*ATko compared to flox/flox controls (1.6- and 1.5-fold, respectively). However, pyruvate tolerance test revealed a moderate decrease in hepatic glucose production of *CGI-58-*ATko mice (Supplementary Fig. 3D) indicating no gross alterations in the gluconeogenic pathway.

### Exogenous FA supply stimulates nuclear CREBH translocation and interferes with the ER stress pathway

**Fig. 3:**
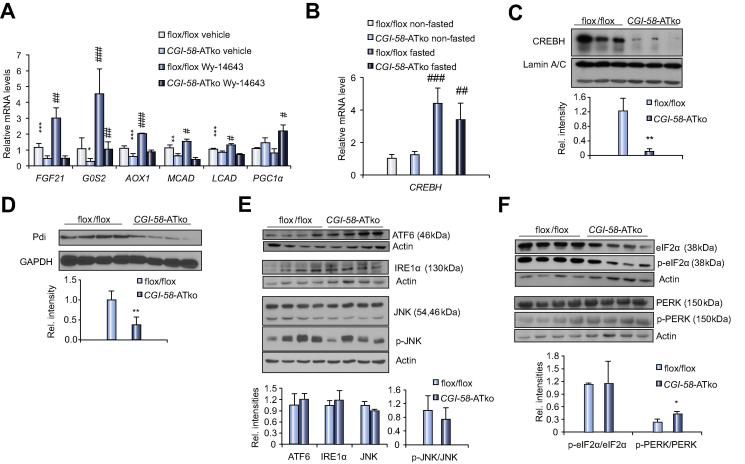
**Defective AT-lipolysis interferes with nuclear CREBH abundance and ER stress gene expression in the liver.** (A) mRNA levels of PPARα-regulated genes in the liver following PPARα agonist (Wy14643) application. (B) Hepatic mRNA expression of *CREBH* in non-fasted compared to fasted mice. (C) CREBH protein level in nuclear extracts prepared from liver tissue of fasted flox/flox and *CGI-58-*ATko mice. CREBH signal intensities were normalized to nuclear Lamin A/C. (D) Pdi protein level in liver tissue obtained from fasted flox/flox and *CGI-58-*ATko mice normalized to GAPDH. (E) Protein levels of the ER stress regulators ATF6, IRE1α, JNK and phosphorylated JNK. Signal intensities were normalized to β-actin and ratio of phosphorylated JNK *vs.* total JNK was calculated. (F) Ratio of phosphorylated eIF2α and phosphorylated PERK *vs.* total protein. Values are mean ± SD from at least 5 mice per genotype (for Western blotting *n *= 3–4). ^*^p <0.05, ^**^p <0.01, and ^***^p <0.001 flox/flox *vs. CGI-58-*ATko; ^*#*^*p *<0.05, ^*##*^*p *<0.01, and ^*###*^*p *<0.001 non-fasted *vs.* fasted or vehicle *vs.* Wy14643.

The changes in hepatic gene expression of *CGI-58-*ATko mice prompted us to further explore the underlying mechanisms. Considering the established role of FAs as PPARα ligands it is conceivable that defective AT-lipolysis restricts ligand availability in the liver. Application of the established PPARα ligand Wy14643 further increased hepatic mRNA levels of PPARα-regulated genes (Fig. 3A) in flox/flox control mice (including a 2.9- and 4.5-fold increase in *FGF21* and *G0S2* expression) whereas mRNA expression of these genes remained low in *CGI-58-*ATko mice. Failed recovery of hepatic gene expression in *CGI-58-*ATko mice suggested an additional ligand-independent defect. Global CREBH-deficiency was shown to markedly impair *FGF21* and *G0S2* mRNA expression in the liver [21], [22] suggesting that these genes are under dual regulation of PPARα and CREBH. Fasting similarly increases *CREBH* mRNA expression in the liver of flox/flox and *CGI-58-*ATko mice (Fig. 3B). CREBH-activated gene expression requires translocation of processed microsomal CREBH to the nucleus. Notably, CREBH protein was substantially reduced (−91%) in nuclear preparations derived from liver tissue of fasted *CGI-58-*ATko compared to flox/flox mice (Fig. 3C) suggesting a defect in CREBH-activated gene expression.

CREBH cleavage and nuclear translocation is known to be induced by fasting [21] and endoplasmic reticulum (ER) stress [23]. It is conceivable that the fasting-induced enhanced flow of exogenous FAs to the liver can be sensed by hepatocyte ER thereby affecting expression of genes linked to the ER stress pathway. Fasting substantially increased mRNA expression of molecular chaperones and ER stress markers (Supplementary Fig. 4A) encompassing glucose-regulated protein 78 (Grp78/BiP), C/EBP homologous protein (Chop), protein disulfide isomerase (Pdi) and ER DnaJ homologue 4 (Erdj4) in the liver of flox/flox mice (ranging from 1.6- up to 3.3-fold). This induction was absent or less pronounced in the liver of fasted *CGI-58-*ATko mice. On the protein level, Pdi signal intensity was significantly reduced (−62%) in *CGI-58-*ATko liver preparations (Fig. 3D) whereas Grp78/BiP protein expression was comparable to flox/flox controls (not shown). To further examine whether fasting interferes with hepatic ER stress we measured protein and/or phosphorylated protein levels of the three ER transmembrane effector proteins of the unfolded protein response (UPR) and their downstream targets including the activating transcription factor-6 (ATF-6), inositol requiring enzyme 1 (IRE1) and PKR-like ER kinase (PERK). ATF-6 protein expression was 1.8-fold increased in liver preparations of fasted *CGI-58-*ATko compared to flox/flox mice whereas IRE1α levels were comparable to flox/flox samples (Fig. 3E). Hepatic JNK protein levels and ratio of phosphorylated to total JNK were unchanged in *CGI-58-*ATko mice compared to flox/flox controls which is in accordance with unchanged mRNA levels of the PPARα co-repressors Ncor1 and Nrip1 (Supplementary Fig. 4B) which are under regulation of JNK [24]. Additionally, ratio of phosphorylated to total PERK was moderately increased in liver preparations of *CGI-58-*ATko mice whereas the ratio of phosphorylated to total eIF2alpha was unchanged (Fig. 3F). Finally, the increase in ATGL expression prompted us to measure protein levels of the forkhead box O1 (FoxO1) transcription factor which has been shown to increase ATGL expression in AT [25]. Insulin stimulates FoxO1-phosphorylation thereby preventing nuclear translocation. Cytosolic FoxO1 protein levels were reduced whereas nuclear levels increased (Supplementary Fig. 4C) indicating that FoxO1 may play a role in the induction of hepatic ATGL expression in fasted *CGI-58-*ATko mice. Plasma levels of alanine aminotransferase (ALT), as an indicator for liver injury, were similar in *CGI-58-*ATko compared to flox/flox mice (23.2 ± 6.3 *vs.* 25.7 ± 9.1).

### Impaired AT-lipolysis counteracts hepatic steatosis, reduces hepatic stress and improves systemic insulin sensitivity of CGI-58-ATko mice on high fat diet

**Fig. 4:**
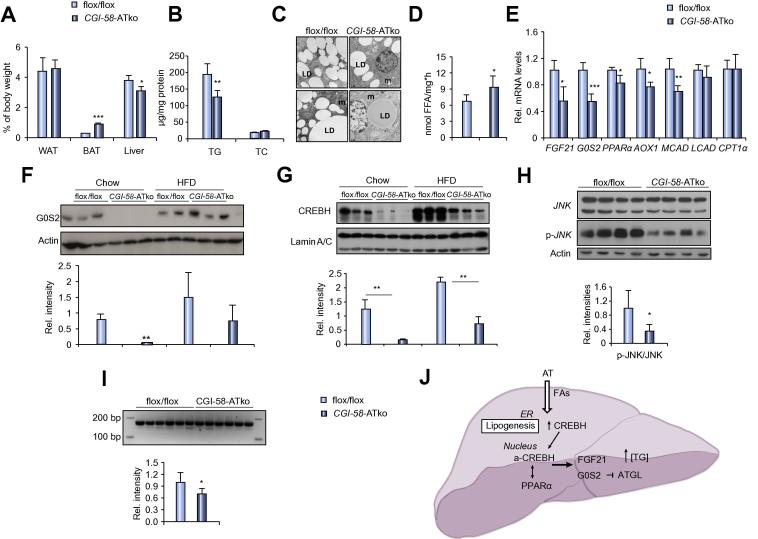
**Impaired AT-lipolysis counteracts hepatic stress and TG accumulation via promoting hepatic TG catabolism on high fat diet.** (A) Organ weights of mice kept on high fat diet (HFD) for 10 weeks starting at the age of 5 weeks. (B) Hepatic TG and TC content upon HFD-feeding in fasted mice. (C) Representative images of hepatic tissue morphology analyzed by transmission electron microscopy (Scale bar = 1 μm; LD, lipid droplet; m, mitochondria; n, nucleus). (D) TG hydrolytic activities in liver tissue of fasted mice. (E) Hepatic mRNA levels of selected PPARα- and CREBH-regulated genes of fasted flox/flox and *CGI-58-*ATko mice determined by qRT-PCR. (F) Hepatic G0S2 and (G) nuclear CREBH protein expression on chow compared to HFD in fasted mice normalized to β-actin and Lamin A/C, respectively (*n *= 3). (H) Ratio of phosphorylated JNK *vs.* total JNK. (I) PCR-amplification of non-spliced and spliced Xbp1 (171 bp and 145 bp, respectively) from hepatic cDNA separated by agarose gel electrophoresis. (J) Simplified scheme depicting the role of AT-derived FAs in the regulation of fasting gene expression in the liver. Values are mean ± SD from at least 5 mice per genotype. ^*^p <0.05, ^**^p <0.01, and ^***^p <0.001 flox/flox *vs. CGI-58-*ATko (on chow or HFD). (This figure appears in colour on the web.)

Feeding a high fat diet (HFD) comparably increased body weight and fat mass of flox/flox and *CGI-58-*ATko mice whereas lean mass was moderately reduced in *CGI-58-*ATko mice (Supplementary Fig. 5A and B). Gonadal AT depots were unchanged whereas brown AT mass was increased and liver mass was reduced in *CGI-58-*ATko compared to flox/flox mice (Fig. 4A). Plasma FA, glucose and insulin levels and HOMA-IR index were lower in *CGI-58-*ATko compared to flox/flox mice (Table 2). Hepatic TG levels were reduced (−37%) in *CGI-58-*ATko mice whereas total cholesterol levels were comparable to flox/flox controls (Fig. 4B). Histomorphological analyses showed the presence of partially large LDs in hepatocytes of both genotypes, whereas LD numbers were 1.6-fold reduced in *CGI-58-*ATko hepatocytes (Fig. 4C; Supplementary Fig. 5C). Hepatic TG hydrolytic activities and ATGL expression were moderately increased in *CGI-58-*ATko compared to flox/flox mice (Fig. 4D; Supplementary Fig. 5D and E) whereas mRNA levels of PPARα-regulated genes (Fig. 4E) and plasma FGF21 concentrations (Table 2) remained low in *CGI-58-*ATko mice on HFD. Similarly, G0S2 protein expression (Fig. 4F) and nuclear abundance of CREBH (Fig. 4G) were reduced in *CGI-58-*ATko mice compared to controls albeit CREBH levels strongly increased in both genotypes on HFD. Next we investigated the impact of HFD on hepatic stress parameters. Notably, reduced plasma ALT levels (Table 2) together with a significant decrease (−65%) in the ratio of phosphorylated to total JNK (Fig. 4H) indicates that impaired AT-lipolysis counteracts the development of hepatic injury and stress. Furthermore, reduced levels of spliced Xbp-1 mRNA (Fig. 4I) indicate less induction of the UPR. However, levels of other proteins from the UPR (Supplementary Fig. 5F–H) were similar in both genotypes except for a moderate reduction in the ratio of phosphorylated *vs.* total PERK and decreased Pdi expression in *CGI-58-*ATko mice. Finally, we investigated the impact of HFD on hepatic insulin signaling. Hepatic ratio of phosphorylated (pSer473) *vs.* total Akt was mildly increased in *CGI-58-*ATko mice on chow whereas the ratio was similar in both genotypes on HFD (Supplementary Fig. 5I).

**Table 2:** **Plasma energy metabolites and hormone levels in fasted flox/flox compared to *CGI-58-*ATko mice on HFD. Plasma insulin and HOMA-IR refer to 6 h fasted mice.**

Values represent mean ± SD; ^*^p <0.05, ^**^p <0.01, ^***^p <0.001 (n ⩾5).

## Discussion

Fasting is associated with increased activity of transcription factors in the liver which are directly or indirectly activated by FAs [18], including PPARs, CREBH and HNF4α. Yet, the role of AT-derived FAs in the regulation of hepatic gene expression is still unclear. In this study we investigated the functional role of AT-derived FAs in the regulation of hepatic gene expression. We found that defective AT-lipolysis in mice, lacking the lipolytic co-activator *CGI-58* (or *ATGL*) in AT, severely impairs PPARα and CREBH co-regulated gene expression in the liver.

Our study demonstrates that hepatic expression of the lipase inhibitor G0S2 and the metabolic regulator FGF21 essentially depend on FA supply from AT. Recently, G0S2 has emerged as an important metabolic regulator [26], [27] via acting as a lipolytic suppressor. G0S2 is inversely expressed in AT and liver depending on the nutritional state: Fasting lowers G0S2 expression in AT to enhance lipolysis and FA supply. In contrast, G0S2 expression increases in the liver upon fasting to suppress TG catabolism thereby preserving hepatic TG as an energy source or pool for lipoprotein biogenesis or signaling molecules. However, the mechanism and metabolites or hormones regulating G0S2 expression in the liver remain unclear. Our study provides several lines of evidence that G0S2 expression is regulated via exogenous FA flow to the liver: i) Defective AT-lipolysis was associated with the absence of fasting-induced G0S2 mRNA expression in the liver which was reversed by raising fasting plasma FA levels; and ii) Defective AT-lipolysis was linked to low nuclear levels of processed active CREBH. This is in line with the severe reduction in hepatic G0S2 expression of mice globally lacking CREBH [21], [22].

Low hepatic G0S2 expression was paralleled by increased TG hydrolytic activities in liver preparations of *CGI-58-*ATko mice which was reduced to control levels by incubation with a specific ATGL inhibitor [13] suggesting that the decline in hepatic TG is mainly due to increased ATGL-mediated TG catabolism. In line with this conclusion, liver-specific G0S2 overexpression leads to hepatic steatosis [26], [28]. Liver-specific G0S2 knockdown does not affect fasting plasma FA levels and leads to increased ketogenesis and FAO gene expression by increasing hepatic TG catabolism [26]. Interestingly, and in strong contrast, impaired hepatic G0S2 expression in *CGI-58-*ATko mice reduced ketone body levels, FAO and ketogenic gene expression indicative for a critical role of AT-derived FAs in the regulation of the hepatic fasting response. Additionally, hepatic glycogen levels increased upon liver-specific G0S2 knockdown whereas glycogen was depleted in fasted *CGI-58-*ATko mice which may be the consequence of restricted availability of FAs as energy fuel in *CGI-58-*ATko mice.

Interestingly, it has been reported that the interaction of the nuclear transcription factors PGC-1α and FOXO1 stimulates gluconeogenesis [29] and that PGC-1α induces gluconeogenesis in an HNF4α-dependent manner [20]. Considering the increased nuclear abundance of the aforementioned transcription factors in fasted *CGI-58-*ATko mice of this study, one would predict increased gluconeogenesis. However, we found that hepatic glucose production was moderately reduced in *CGI-58-*ATko mice in contrast to mice with hepatic G0S2 knock down, which increases hepatic glucose production in pyruvate tolerance tests. Hepatic glucose formation in the fasted state also involves the utilization of AT-derived glycerol as metabolite and glycogenolysis. It is conceivable that lowered glucose production in response to pyruvate administration in fasted *CGI-58-*ATko mice involves the deprivation of glycerol and glycogen and possibly restricted FA supply as energy fuel.

Notably, the induction of hepatic TG-breakdown either in mice globally lacking G0S2 or in mice lacking CGI-58 in AT that were fed a HFD increased systemic insulin sensitivity. Furthermore, both mutant mouse models exhibited similarly reduced plasma ALT levels demonstrating that the stimulation of hepatic TG-breakdown *per se* counteracts the development of metabolic disorders. Similar to impaired G0S2 expression, we show that the induction of hepatic FGF21 expression depends on AT-FA supply. FGF21 expression not only stimulates ketogenesis but also regulates cold adaptation and systemic energy metabolism. The severe defect in cold adaptation observed in fasted *CGI-58-*ATko mice further demonstrates the critical role of AT-derived FAs in the induction of hepatic FGF21 expression and the regulation of whole body energy homeostasis [19]. Interestingly, hepatic FGF21 expression and serum FGF21 levels have been reported to be significantly increased in obese patients [30] and may involve changes in exogenous FA levels.

In this study we found that PPPAα-ligand treatment of *CGI-58-*ATko mice did not recover PPARα-regulated gene expression in the liver suggesting a PPARα ligand-independent defect. Recently Kim *et al.* [31] showed that a mutual transcriptional regulation exists between CREBH and PPARα and that PPARα requires CREBH for *trans*-activation of *FGF21* gene expression. The extremely low protein levels of processed nuclear CREBH in fasted *CGI-58-*ATko mice suggested that AT-derived FAs are an important energy sensor for the liver, required for CREBH cleavage and nuclear translocation to activate *G0S2* and *FGF21* gene expression. CREBH was originally discovered as an ER stress regulated gene, albeit its role in ER stress is a matter of discussion [32]. Impaired AT-lipolysis divergently interfered with protein expression and/or activation of the three major regulators of the UPR pathways, including increased ATF6 protein expression and PERK-phosphorylation in *CGI-58-*ATko mice on chow, implicating that low nuclear abundance of CREBH was not due to classic induction of hepatic ER stress and involves a currently unknown mechanism.

It is conceivable that the increase in LD-formation in the fasted state (which is impaired in *CGI-58-*ATko mice) is sensed by the ER and triggers CREBH cleavage and nuclear translocation which is required for PPARα- and CREBH-coordinated expression of G0S2 and FGF21 (Fig. 4J) to regulate hepatic and whole body TG homeostasis. The fasting-stimulated induction of G0S2 expression may protect the liver from excessive TG catabolism which preserves TG as energy fuel and reservoir for lipoprotein-TG synthesis under normal diet. Under conditions of increased plasma FA levels, as exemplified in mice fed a HFD, a sustained G0S2-mediated suppression of hepatic TG catabolism may lead to the development of hepatic steatosis and the activation of the UPR. In line with such an assumption, reduced plasma FA levels of *CGI-58-*ATko mice on HFD were accompanied by increased hepatic TG-breakdown, reduced levels of phosphorylated JNK and PERK together with decreased mRNA levels of spliced *Xbp-1* in liver tissue of *CGI-58-*ATko mice. Additionally, reduced insulin levels and HOMA-IR in *CGI-58-*ATko mice on HFD suggest that the reduction in circulating FA levels due to impaired AT-lipolysis counteracts the development of whole body and possibly hepatic insulin resistance.

Together, in this study we show that induction of G0S2 and FGF21 expression in the liver depends on FA supply from AT and that plasma FA levels correlate with hepatic G0S2 expression and nuclear abundance of CREBH. Accordingly, a surplus of plasma FAs, linked to obesity and/or dietary fat content, may promote hepatic fat accumulation via sustained suppression of hepatic TG catabolism. Impaired hepatic TG catabolism leads to hepatic steatosis in humans and mice [5], [6], [33]. Inhibition of AT-lipolysis or hepatic G0S2 expression may represent an effective strategy to enhance hepatic TG catabolism thereby counteracting hepatic fat accumulation and NAFLD development.

## Financial support

This research was supported by the grants 10.13039/501100002428FWF P20602-B05, DK-MCD W1226, P25193, P 25944-B19, J3221-B19 and SFB Lipotox F30-B05 funded by the 10.13039/501100002428Austrian Science Fund and the 10.13039/100000002National Institutes of Health grant R01-DK-090166 and a Howard Hughes Medical Institute Physician-Scientist Early Career Award (to E.E.K.).

## Conflict of interest

The authors who have taken part in this study declared that they do not have anything to disclose regarding funding or conflict of interest with respect to this manuscript.

## Author contributions

D. Jaeger and G. Haemmerle designed the study and were involved in all aspects of the experiments and wrote the manuscript. G. Schoiswohl performed studies with *ATGL-*ATko mice. M. Schweiger, Renate Schreiber, Nadja Poecher, T. Eichmann, P. Hofer, N. Pollak, R. Schreiber, F. Radner, G. Grabner, and K. Zierler contributed to experiments and acquisition of data. S. Eder guided animal handling. D. Kolb performed electron microscopy. K. Preiss-Landl, A. Lass, R. Zechner, and E. Kershaw discussed the results.
