# Supplementary material for: Fasting-induced G0/G1 switch gene 2 and FGF21 expression in the liver are under regulation of adipose tissue derived fatty acids
Source: J Hepatol. 2015 Aug;63(2):437–45. doi: 10.1016/j.jhep.2015.02.035 (PMC4518503; doi:10.1016/j.jhep.2015.02.035)
Supplement: Supplementary data [file mmc1.pdf]

Supplementary data to:

Fasting-induced G0/G1 switch gene 2 and FGF21 expression in the liver are under regulation of adipose tissue derived fatty acids

Doris Jaeger[1], Gabriele Schoiswohl[2], Peter Hofer[1], Renate Schreiber[1], Martina Schweiger[1], Thomas O. Eichmann[1], Nina M. Pollak[1], Nadja Poecher[1], Gernot F. Grabner[1], Kathrin A. Zierler[1], Sandra Eder[1], Dagmar Kolb[3], Franz P. W. Radner[1], Karina Preiss-Landl[1], Achim Lass[1], Rudolf Zechner[1], Erin E. Kershaw[2], and Guenter Haemmerle[1§]

Table of Contents

Supplementary experimental procedures 3
- Animals and diets 3
- Gene expression analysis by quantitative real-time PCR 4
- Xbp1-PCR 4
- Plasma parameters and HOMA-IR....................4
- Plasma ALT 5
- Plasma non-saturated and saturated FA species....................5
- Measurement of tissue TG hydrolytic activities 5
- Measurement of tissue TG levels....................6
- Measurement of glycogen in liver tissue 7
- Pyruvate tolerance tests 7
- Western Blot analyses 7
- Acute cold exposure 9

Supplementary figures....................11
- Supplementary Fig. 1:....................11
- Supplementary Fig. 2:....................13

Supplementary Fig. 3 15
Supplementary Fig. 4 16
Supplementary Fig. 5 17
Supplementary Tables 19
Supplementary table 1 19
Supplementary table 2 20
Supplementary references 22

SUPPLEMENTARY EXPERIMENTAL PROCEDURES

Animals and diets

CGI-58-floxed mice [1,2] and *Fab4*-Cre transgenic mice [3] have been previously described and used for the generation of mice with AT-selective disruption of CGI-58 (CGI-58ATko). Littermates homozygous for the floxed CGI-58 allele (flox/flox) were used as controls. *Atgl*-floxed mice [4] and *AdipoQ*-Cre transgenic mice [5] have been used for the generation of mice with AT-selective disruption of ATGL (ATGL-ATko). Mice were housed in a pathogen-free animal facility on a regular 12-h light/dark cycle and had free access to food and water. 14- to 16-week-old controls and CGI-58ATko mice were fed a standard chow diet (Ssniff) or subjected to a high fat diet containing 45% calories from fat (Ssniff) starting at 5 weeks of age for the duration of 10 weeks. Animals were subjected to 6-h-fasting or overnight-fasting (16-h-fasting) and then sacrificed.

For the administration of the PPARα agonist Wy14643 (Cayman Chemical), 12-h-fasted control and CGI-58ATko mice were i.p. injected either the Wy14643 compound (50 µg/g body weight in 50% DMSO) or equivalent amounts of vehicle (50% DMSO in 0.9% NaCl). After another 2-h-fasting period, mice were sacrificed and tissues were excised.

Animal care and study protocols were approved by the Austrian ethics committee and were in accordance with the Council of Europe Conventions.

For raising plasma FA levels, mice were fasted for 12 h and were then administered an intragastric olive oil gavage (200 µl/mouse). To release lipoprotein lipase (LPL) from the luminal site of capillaries and to hydrolyze TG-rich lipoproteins, heparin was administered *i.p.* (10 IU/mouse). 2 h after oil-gavage/heparin, mice were anaesthetized

and blood was collected from the retro-orbital sinus for subsequent plasma analysis. Thereafter, mice were euthanized by cervical dislocation and tissues were harvested and snap-frozen until further qRT-PCR analysis.

Gene expression analysis by quantitative real-time PCR

Total RNA was isolated with TRIzol reagent (Invitrogen) and treated with DNase I (Invitrogen). One µg total RNA was reverse transcribed using the MultiScribe High Capacity Reverse Transcription kit (Applied Biosystems) at 37°C for 2 hours. Quantitation of relative expression was performed using StepOnePlus Real-time PCR system (Applied Biosystems) with denaturation at 95°C and annealing/extension at 60°C over 40 cycles. PCR reactions (20 µl) contained 8 ng cDNA, 10 pmol of each primer and 10 µl Maxima SYBR Green/ROX Master Mix (Thermo Fisher Scientific). Relative mRNA levels were quantified using the ΔΔCt method with ß-actin (non-AT) or 36B4 (AT) as reference gene. Primer sequences are listed in Table S1.

Xbp1-PCR

1µl of liver cDNA was used as template and the PCR was performed applying 3 min 94°C, 30 cycle of 30 sec at 94°C, 30 sec at 58°C, 30 sec at 72°C and finally 3 min at 72°C. PCR products were run on a 3% agarose gel.

Plasma parameters and HOMA-IR

Blood samples were collected from shortly anesthetized non-fasted, 6-h- and 16-h-fasted mice by retro-orbital puncture. Plasma TG, FA, glycerol, hormone and cytokine levels were determined using commercially available kits. The homeostasis model for

insulin resistance (HOMA-IR) was calculated from 6-h-fasting glucose (mmol/L) x fasting plasma insulin (µU/ml) divided by 22.5.Plasma levels for insulin, FGF21, leptin, ketone bodies and levels of plasma growth hormone were analyzed using ELISA or Colorimetric Assay kits from Crystal Chem. (Rat Insulin ELISA kit), Cayman Chemical (ß-Hydroxybutyrate), Millipore/Merk (Rat/Mouse FGF-21 ELISA). Adiponectin levels were determined using the Mouse/Rat Adiponectin ELISA Kit (B-Bridge International, Inc.,CA, USA). Mouse TNF alpha and IL-6 were measured using the ELISAs “Ready-SET-Go!” from eBioscience.

Plasma ALT

ALT activity was determined in plasma of mice using the Infinity ALT(GPT) Liquid stable reagent according to the manufacturer’s protocol. Briefly, 20 µl of plasma were incubated with 200 µl of ALT reagent for a total time of 10 min at 37°C in a Beckman DU640 spectrophotometer. The reaction is monitored by measuring the rate of the decrease in absorbance at 340 nm per minute.

Plasma non-saturated and saturated FA species

Analysis of plasma FA species from overnight fasted control and CGI-58ATko mice was performed as previously described [6].

Measurement of tissue TG hydrolytic activities

Tissue neutral TG hydrolase activity in the absence and presence of recombinant CGI-58 was performed as previously described [7,8]. Briefly, tissue of 16-h-fasted animals was homogenized in Solution A (0.25 M sucrose, 1 mM EDTA, 1 mM DTT, 1 µg/ml

pepstatin, 2 µg/ml antipain, 20 µg/ml leupeptin, pH 7.0) on ice using an Ultra Turrax (IKA) and the infranatant after centrifugation at 20,000 x g and 4°C for 30 min was assayed for TG hydrolase activity. A micellar substrate of phospholipid-emulsified triolein (1.67 mM) containing [9,10-^{3}H]-labeled triolein (Perking Elmer) was used. Tissue lysates in a total volume of 100 µl solution A were incubated with 100 µl substrate in a shaking water bath at 37°C for 60 min in the absence or presence of the ATGL-specific inhibitor ATGListatin (20 µM) [9]. The reaction was terminated by the addition of 3.25 ml methanol/chloroform/n-heptane (10/9/7, v/v/v) and 1 ml 0.1 M potassium carbonate/0.1 M boric acid, pH 10.5. After centrifugation, radioactivity in the upper phase was determined by liquid scintillation counting.

Measurement of tissue TG levels

Tissue lipids were extracted according to the method of Folch et al [10]. Tissue lipid extracts were dried using a SpeedVac (Heto) and re-solubilized by brief sonication in 2 % Triton X-100 (Misonix Ultrasonic Liquid Processor, QSonica). TG concentrations were measured with the Infinity triglycerides kit (Thermo Electron Corporation) using a glycerol standard solution (Sigma). Total cholesterol was measured using cholesterol CHOD-PAP reagent (Roche Diagnostics, Mannheim, Germany) with either the Glycerol Standard Solution (Sigma, St. Louis, MO) or a 2mg/ml cholesterol solution as standard. Remaining tissue pieces were lysed in lysis buffer (0.3 N NaOH/0.1 % SDS) and protein concentration was determined using the BCA Protein Assay Kit (Pierce, Thermo Scientific).

Measurement of glycogen in liver tissue

Snap-frozen tissue was homogenized in 0.03M HCl (20 µl 0.03 M HCl/mg tissue). For the determination of total glucose concentration an aliquot of the tissue homogenate was incubated with equal volumes of 2 M HCl for 2 hours at 90°C. Subsequently, the homogenate was neutralized with 2 M NaOH. For the measurement of free glucose concentration an aliquot of the tissue homogenate was mixed with equal volumes of 2 M HCl and immediately neutralized with 2 M NaOH. After centrifugation of samples for 5 minutes at 10,000 x rpm an aliquot of the supernatant was used for the measurement of glucose concentrations using the D-Glucose-HK Kit (Megazyme, Wicklow, Ireland). The difference between total and free glucose concentrations represented the tissue glycogen content.

Pyruvate tolerance tests

After an overnight fast (16 h), mice were injected with sodium-pyruvate (2g/kg body weight) dissolved in saline and glucose blood glucose levels were determined after 15, 30, 60, 90 and 120 min.

Western Blot analyses

Tissues of 16-h-fasted mice were homogenized in Solution A (0.25 M sucrose, 1 mM EDTA, 1 mM DTT, 1 µg/ml pepstatin, 2 µg/ml antipain, 20 µg/ml leupeptin, pH 7.0) on ice using an Ultra Turrax (IKA). Tissue lysates were centrifuged at 20,000 x g and 4°C for 30 min and the infranatants were assayed for protein concentration using the Bio-Rad protein assay (Bio-Rad Laboratories) and BSA as standard.

Nuclear extracts were prepared from frozen liver samples either by the use of the NE-PER Nuclear and Cytoplasmic Extraction Reagents following the manufacturer's instructions (Pierce, Thermo Scientific) or by using a protocol modified from Lamond Lab Protocol. Briefly, tissue pieces were homogenized in Buffer A (10 mM HEPES pH 7.9, 1.5 mM MgCl2, 10 mM KCl, 0.5 mM DTT, 1 µg/ml pepstatin, 2 µg/ml antipain, 20 µg/ml leupeptin) using a Wheaton tissue grinder (Thermo Scientific). The homogenate was filtered through a cell strainer (70 µm, Corning) and centrifuged at 228 x g and 4°C for 10 min. The supernatant was withdrawn containing cytosolic proteins. Subsequently, the pellet was resuspended in Buffer A containing 1 % NP-40 using a tissue grinder and centrifuged at1,000 x g and 4°C for 10 min. After rinsing in Buffer A, the pellet was resuspended in sucrose solution S1 (0.25 M sucrose, 10 mM $MgCl_2$) and layered over sucrose solution S3 (0.88 M sucrose, 0.5 mM $MgCl_2$). After centrifugation at 2,800 x g and 4°C for 10 min, the nuclear pellets were sonicated twice for 10 seconds in RIPA buffer (50 mM Tris pH 7.5, 150 mM NaCl, 1 % NP-40, 0.5 % deoxycholate, 1 µg/ml pepstatin, 2 µg/ml antipain, 20 µg/ml leupeptin) using a Misonix Ultrasonic Liquid Processor (QSonica). The supernatant obtained after centrifugation at 2,800 x g and 4°C for 10 min was collected as nuclear extract and assayed for protein concentration using the BCA Protein Assay Kit (Pierce, Thermo Scientific) and BSA as standard.

Equivalent amounts of protein homogenates and nuclear extracts were separated by SDS-PAGE according to standard protocols and blotted onto a polyvinylidene fluoride membrane (Carl Roth GmbH). Blots were probed with antibodies raised against CGI-58 (designated as ABHD5, Abnova), GAPDH (Cell Signaling), ATGL (Cell Signaling), G0S2 (kindly provided byS. Kersten) [11], CREBH (kindly provided by A.-H. Lee) [12], Lamin A/C (Santa Cruz Biotechnology), BiP (Cell Signaling), Pdi (Cell Signaling), HNF-4α

(Santa Cruz Biotechnology), HDAC2 (Invitrogen, Life technologies), PGC-1α (Calbiochem, Millipore), Total FoxO1 (Cell Signaling), ATF6 (NBP1-40256, Novus Biologicals, Littleton, CO), eIF2α and phosphorylated-eIF2 α (Cell Signaling Technology, Danvers, MA), JNK and phosphorylated-JNK (Cell Signaling), IRE1α (Cell Signaling), PERK and phosphorylated PERK (Cell Signaling Technology), and mouse monoclonal antibody raised against β-actin (Santa Cruz Biotechnology, Dallas, TX). Specifically bound immunoglobulins were detected in a second reaction using horseradish peroxidase-conjugated anti-rabbit or anti-mouse IgG antibodies (1:10,000 dilution) and visualized by enhanced chemiluminescence detection (ECL, Pierce/Thermo Scientific, Waltham, MA).

For analysis of hepatic insulin sensitivity, 6-h fasted mice were injected with insulin (1U/kg body weight) and after 10 min livers were dissected for determination of phosphorylated (pSer473) and total Akt using antibodies from EMD Millipore. Levels were normalized to ß-Actin and pAkt/Akt ratios were calculated.

Bound immunoglobulins were detected using horseradish peroxidase-conjugated anti-rabbit, anti-mouse or anti-goat IgG antibody (Vector laboratories, GE Healthcare, Millipore) and visualized by enhanced chemiluminescence detection (ECL Plus Western blotting substrate, Pierce, Thermo Fisher Scientific). Quantification of signal intensities was performed using the ImageJ software.

Acute cold exposure

For acute cold exposure experiments, non-fasted and 10 hours fasted mice were single-housed and exposed to 4°C. Before the start of the experiment and at indicated time points rectal body temperature was measured by the usage of a Temp10T

Thermocouple thermometer (Thermo Scientific, Waltham, MA) and a Ret-3 rectal probe for mice (Physitemp, Clifton, NJ).

SUPPLEMENTARY FIGURES

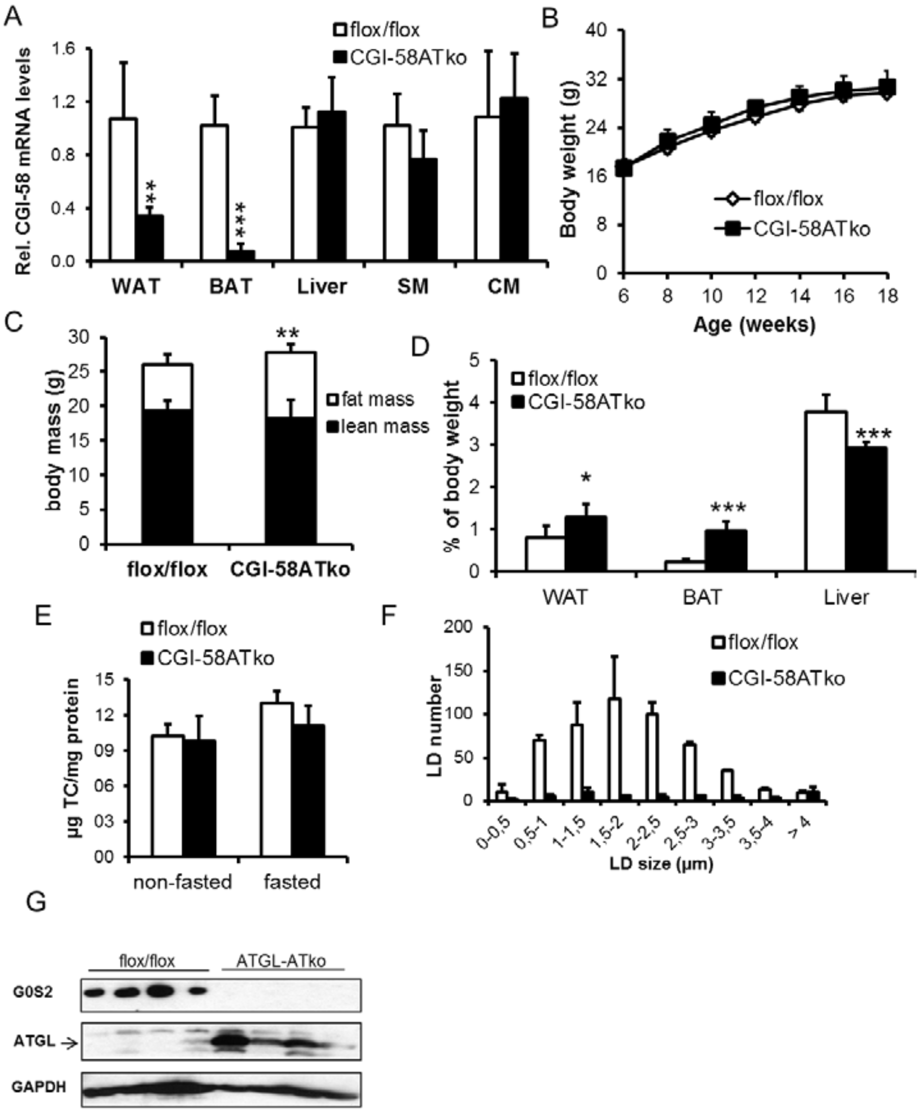

Supplementary Fig. 1

***Supplementary Fig. 1:* (A)** *CGI-58 mRNA expression, body analyses, liver total cholesterol and LD analysis in fasted CGI-58ATko compared to flox/flox mice.* (A) In line with CGI-58 protein expression (Fig. 1A), CGI-58 mRNA levels were markedly and exclusively reduced in gonadal white and brown AT (WAT and BAT) of fasted CGI-58ATko compared to flox/flox mice. (B) Bodyweight monitored over a period of 18 weeks starting at the age of 6 weeks. (C) Lean body

mass and fat mass of 15-week old mice. (D) Organ weights indicated as % of body weight of 15-week old mice. (E) Total cholesterol levels in the liver of non-fasted compared to fasted mice. (F) LD size and numbers were determined from a total area of 18478 µm2 derived from sections of three mice and genotype. In total, 1019 LDs were counted for flox/flox and 111 LDs for CGI-58ATko mice, respectively. (G) Hepatic G0S2 and ATGL protein expression in fasted ATGL-ATko mice compared to controls. Values are mean ± SD from at least 5 mice per genotype. *p <0.05; **p <0.01; ***p <0.001.

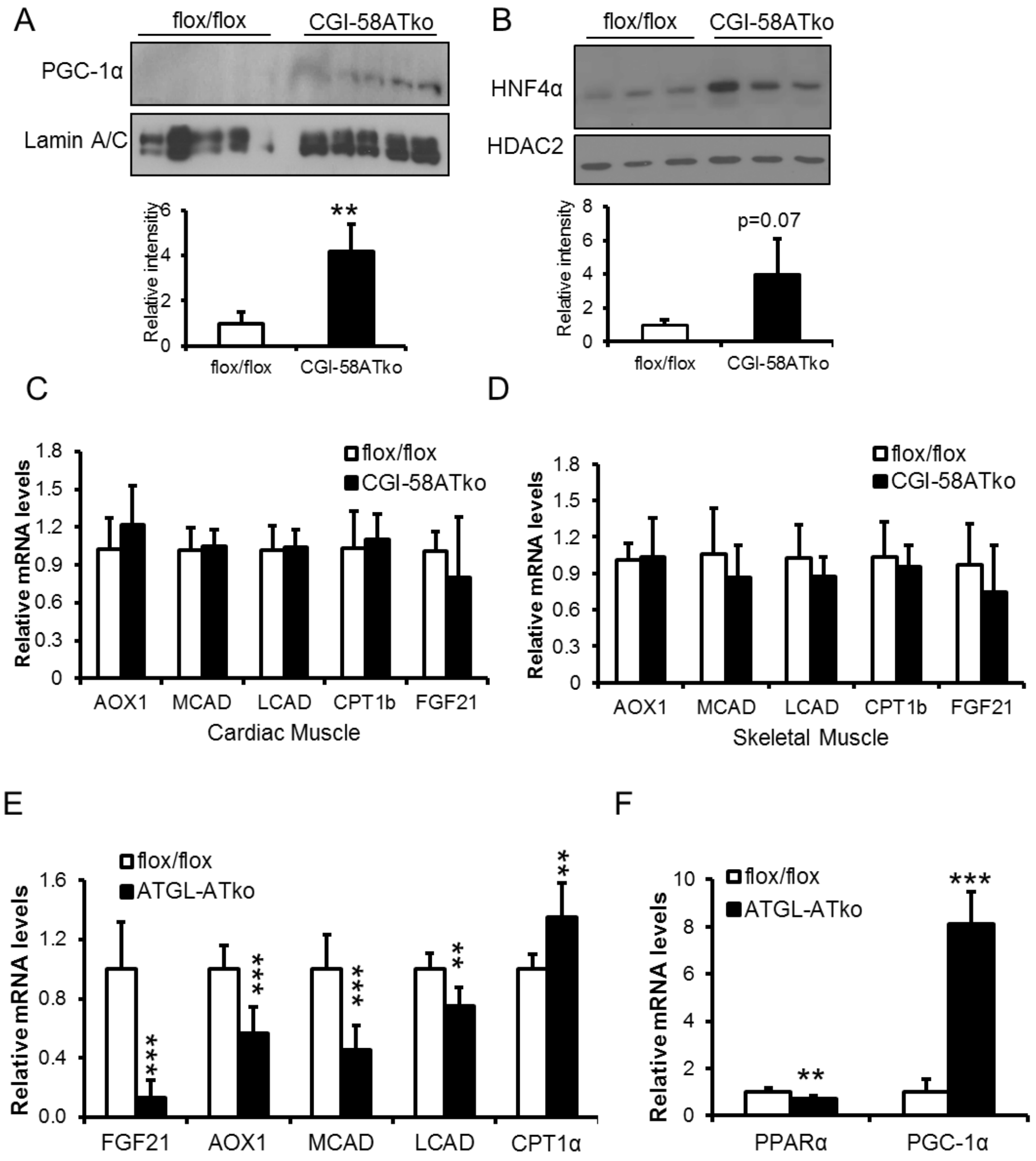

Supplementary Fig. 2

Supplementary Fig. 2: *Hepatic nuclear PGC-1α and HNF4α protein level, PPARα-regulated gene expression in muscle of CGI-58ATko mice and hepatic expression of PPARα-regulated genes in mice lacking ATGL exclusively in AT.* Western blot analysis of (A) PGC-1α and (B) HNF4α protein content in nuclear extracts obtained from liver tissue of fasted mice. For quantitation (lower panels) blots were scanned and bands were analyzed with ImageJ software. (C) Cardiac and (D) skeletal muscle (m. quadriceps) mRNA levels of PPARα-regulated genes in

fasted flox/flox and CGI-58ATko mice. mRNA expression of (E) PPARα-regulated genes, (F) PPARα and PGC-1α in the liver of fasted flox/flox mice compared to levels in mice lacking ATGL exclusively in AT (ATGL-ATko). Values are mean ± SD from at least 5 mice per genotype. $^{**}p < 0.01$; $^{***}p < 0.001$.

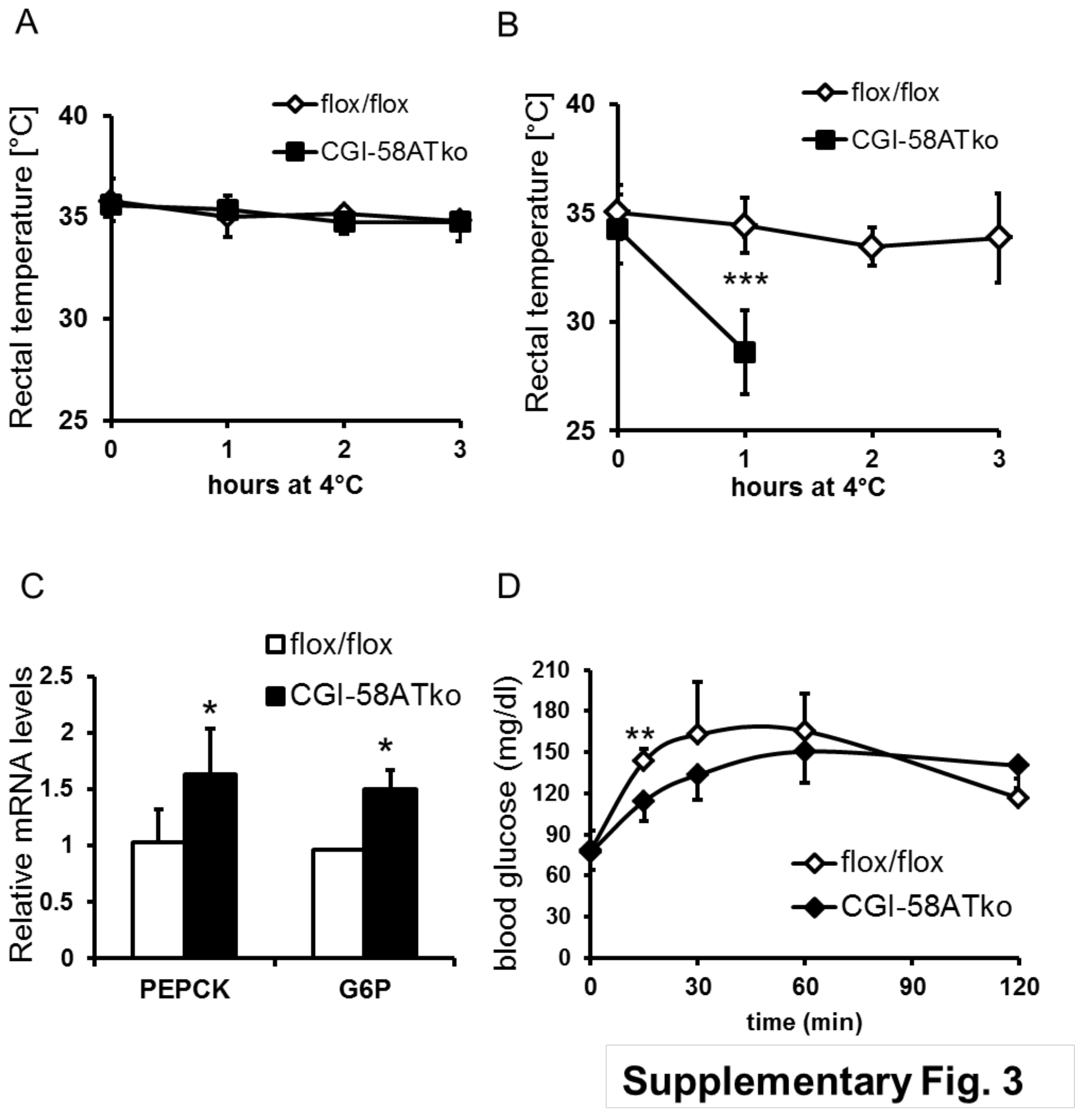

Supplementary Fig. 3*: Cold adaptation, gluconeogenic gene expression and pyruvate tolerance test in fasted mice.* Measurement of body temperature of (A) non-fasted and (B) fasted mice maintained at 4°C. The rapid drop in body temperature of CGI-58ATko mice prompted us to terminate the experiment. (C) mRNA expression levels of phosphoenolpyruvate carboxykinase (PEPCK) and glucose-6-phosphatase (G6P) in the liver of fasted mice determined by qRT-PCR. (D) Pyruvate tolerance test in fasted mice. Glucose was measured before and after the injection of sodium-pyruvate at a dose of 2g/kg body weight. Values are mean ± SD from at least 5 mice per genotype. *p <0.05; ***p <0.001.

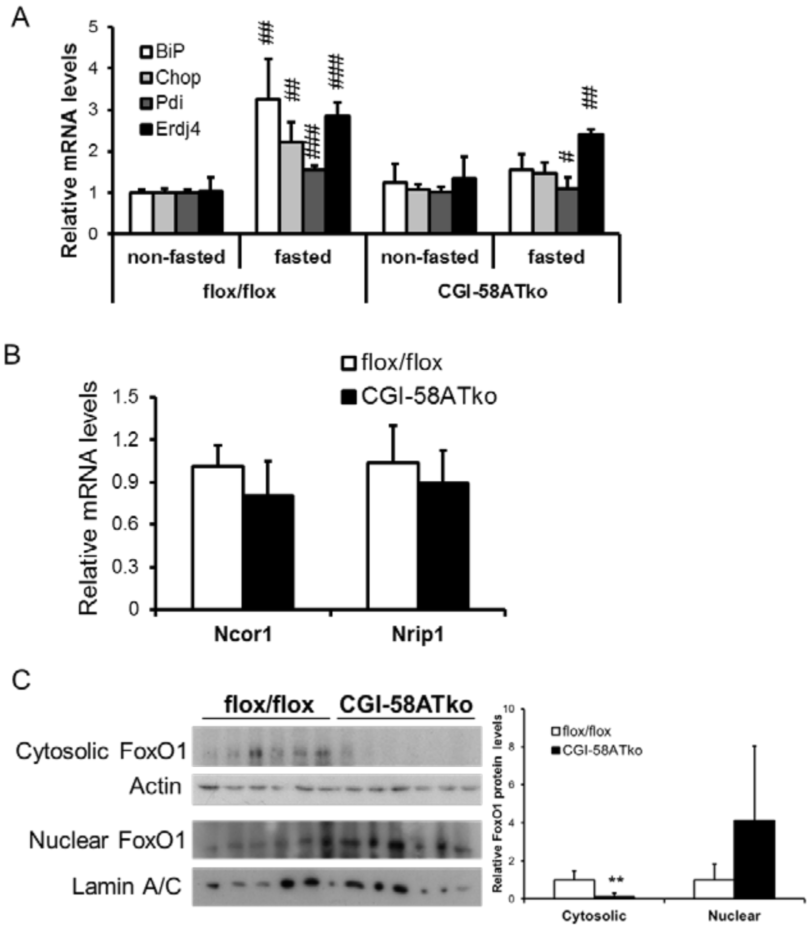

Supplementary Fig. 4

***Supplementary Fig. 4**: Liver mRNA expression of molecular chaperons, proteins from the UPR, nuclear co-repressors and protein levels of FoxO1 nuclear transcription factor.* (A) mRNA expression of selected ER stress marker genes in the liver of non-fasted and overnight-fasted flox/flox and CGI-58ATko mice normalized to levels of non-fasted flox/flox mice. (B) Hepatic mRNA expression levels of the PPARα co-repressors Ncor1 and Nrip1 which are under the regulation of JNK. (C) Measurement of cytosolic FoxO1 protein levels compared to nuclear levels of the transcription factor. Blots were quantitated applying ImageJ software. Values are mean ± SD from at least 5 mice per genotype. $^{\#}p$ <0.05; $^{\#\#}p$ <0.01 $^{\#\#\#}p$ <0.001 depicts non-fasted versus fasted. **p <0.01 flox/flox versus CGI-58ATko.

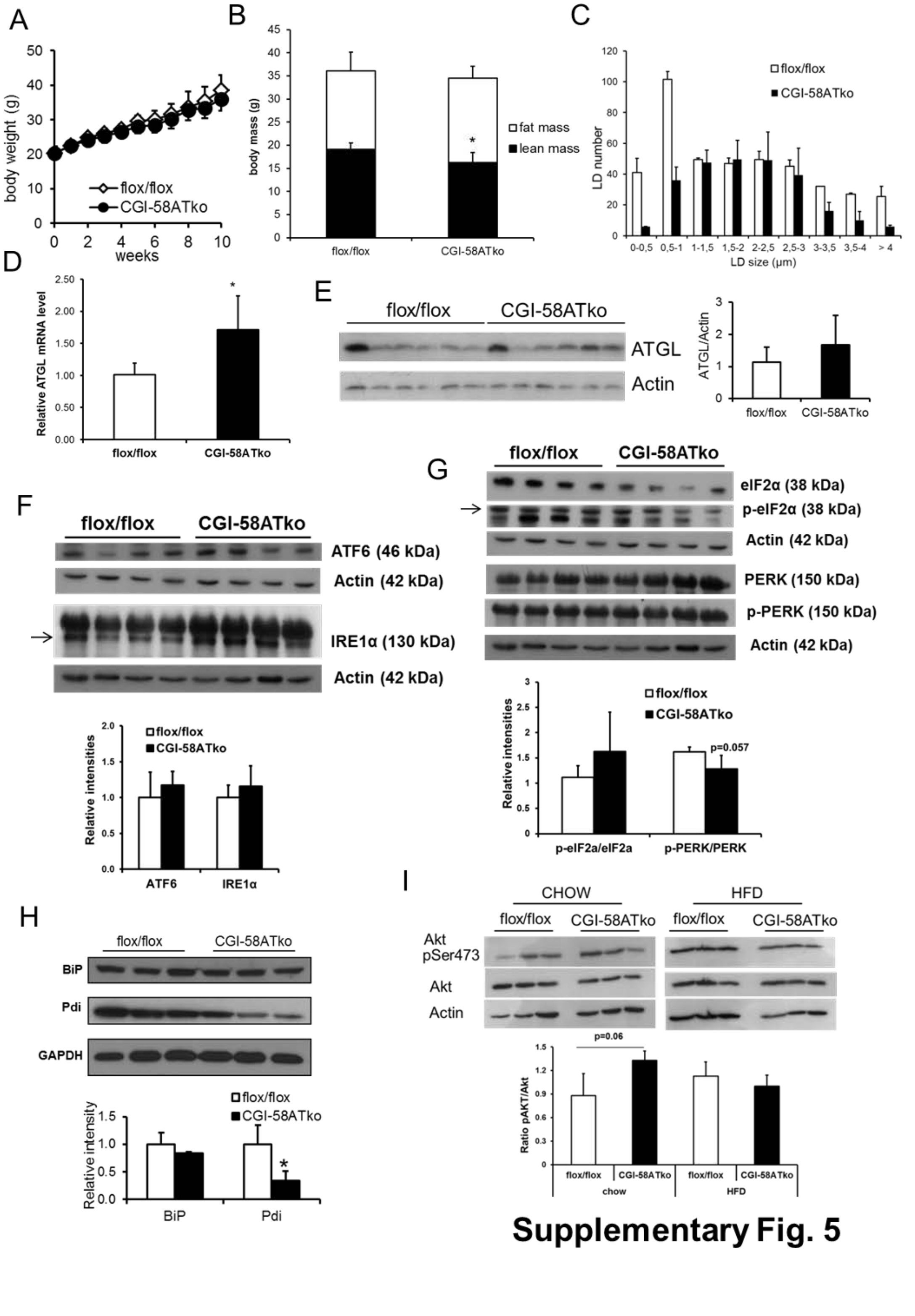

Supplementary Fig. 5

***Supplementary Fig. 5**: Body and LD analyses, TG catabolism and protein levels related to the UPR pathway and insulin signaling on HFD feeding.* Body weight (A) and body mass

composition (B) of mice on HFD for 10 weeks. (C) LD size and numbers were determined from at total area of 19418 µm2 and 18165 µm2 of three flox/flox and CGI-58ATko mice, respectively. In total, 836 LDs were counted for flox/flox and 519 LDs for CGI-58ATko mice, respectively. Hepatic ATGL mRNA (D) and protein (E) expression of fasted mice on HFD. (F) Hepatic protein levels of the ER stress regulators ATF6 and IRE1α normalized to β-actin. (G) Hepatic protein levels of phosphorylated eIF2alpha and PERK versus total. (H) Hepatic protein levels of BiP and Pdi normalized to GAPDH. (I) Protein levels of Akt and phospho-Akt in liver tissue of flox/flox controls and CGI-58ATko mice kept on chow (left panel) or HFD (right panel). Ratio of phosphorylated Akt versus total Akt was calculated. Values are mean ± SD from at least 5 mice per genotype except for western blotting where $n = 3 - 4$. *p <0.05; ***p <0.001.

SUPPLEMENTARY TABLES

Supplementary table 1

Plasma cytokine and adiponectin levels in fasted flox/flox and CGI-58ATko mice on HFD.

	flox/flox	CGI-58ATko
TNFα [pg/ml]	8.1 ± 2.2	3.4 ± 2.3*
IL-6 [pg/ml]	5.1 ± 3.1	3.0 ± 1.9
Adiponectin [µg/ml]	7.6 ± 0.9	7.2 ± 0.9

Values represent mean ± SD; *p< 0.05, **p< 0.01, ***p< 0.001 (*n* = 6).

Supplementary table 2

PCR primer sequences used for qRT-PCR.

Gene	forward primer (5'-3')	reverse primer (5'-3')
ß-actin	AGCCATGTACGTAGCCATCCA	TCTCCGGAGTCCATCACAATG
36B4	GCTTCATTGTGGGAGCAGACA	CATGGTGTTCTTGCCCATCAG
CGI-58[a]	GGTTAAGTCTAGTGCAGC	AAGCTGTCTCACCACTTG
ATGL[b]	GAGACCAAGTGGAACATC	GTAGATGTGAGTGGCGTT
HSL	GCTGGGCTGTCAAGCACTGT	GTAACTGGGTAGGCTGCCAT
PPARα[c]	GTACCACTACGGAGTTCACGCAT	CGCCGAAAGAAGCCCTTAC
PGC-1α[c]	CCCTGCCATTGTTAAGACC	TGCTGCTGTTCCTGTTTTC
CPT1α	CACCAACGGGCTCATCTTCTA	CAAAATGACCTAGCCTTCTATCGAA
CPT1ß	CGAGGATTCTCTGGAACTGC	GGTCGCTTCTTCAAGGTCTG
AOX1	AGATTGGTAGAAATTGCTGCAAAA	ACGCCACTTCCTTGCTCTTC
MCAD	GCAACTGCCCGCAAGTTT	TACTCCCCGCTTTTGTCATATTC
LCAD[b]	TTTCCGGGAGAGTGTAAGGA	ACTTCTCCAGCTTTCTCCCA
FGF21	TCCAAATCCTGGGTGTCAAA	CAGCAGCAGTTCTCTGAAGC
G0S2	TAGTGAAGCTATACGTGCTGGGC	CCGTGGCGGCTGTGAAAGGGT
PEPCK	CAT ATG CTG ATC CTG GGC ATA AC	CAA ACT TCA TCC AGG CAA TGT C
G6P	CCT CCT CAG CCT ATG TCT GC	AAC ATC GGA GTG ACC TTT GG
CREBH[d]	GGCCATTGACCTGGACATGT	TTCACAGTGAGGTTGAAGCGG
BiP[e]	GTTTGCTGAGGAAGACAAAAAGCTC	CACTTCCATAGAGTTTGCTGATAAT
Chop[e]	GTCCAGCTGGGAGCTGGAAG	CTGACTGGAATCTGGAGAG
Pdi[e]	CAAGATCAAGCCCCACCTGAT	AGTTCGCCCCAACCAGTACTT
Erdj4[f]	GTGGAGAAGCTGCGTCGGGG	TGAGGCAGACTTTGGCACACCT
Ncor1	AGAACTTCTGATGTTTCTTCCAG	CTGGAGACTTGGCTGGTATA
Nrip1	CCCCAGTACCAACAGGACTACC	TGAACGTGGCGGAATTTTGT
HMGCS2	AGAGAGCGATGCAGGAAACTT	AAGGATGCCCACATCTTTTGG
Xbp1	ACACGCTTGGGAATGGACAC	CCATGGGAAGATGTTCTGGG

Table footnotes:

[a] Zierler, K.A., Jaeger, D., Pollak, N.M., Eder, S., Rechberger, G.N., Radner, F.P.W., Woelkart, G., Kolb, D., Schmidt, A., Kumari, M., et al. (2013). Functional cardiac lipolysis in mice critically

depends on comparative gene identification-58. The Journal of Biological Chemistry 288, 9892–9904

[b] Haemmerle, G., Moustafa, T., Woelkart, G., Büttner, S., Schmidt, A., Van de Weijer, T., Hesselink, M., Jaeger, D., Kienesberger, P.C., Zierler, K.A., et al. (2011). ATGL-mediated fat catabolism regulates cardiac mitochondrial function via PPAR-α and PGC-1. Nature Medicine 17, 1076-1085.

[c] Murholm, M., Dixen, K., Qvortrup, K., Hansen, L.H., Amri, E.Z., Madsen, L., Barbatelli, G., Quistorff, B., and Hansen, J.B. (2009). Dynamic regulation of genes involved in mitochondrial DNA replication and transcription during mouse brown fat cell differentiation and recruitment. PLoS One 4, e8458.

[d] Lee, J.H., Giannikopoulos, P., Duncan, S.A., Wang, J., Johansen, C.T., Brown, J.D., Plutzky, J., Hegele, R.A., Glimcher, L.H., and Lee, A.H. (2011). The transcription factor cyclic AMP-responsive element-binding protein H regulates triglyceride metabolism. Nature Medicine 17, 812-815.

[e] Kawasaki, N., Asada, R., Saito, A., Kanemoto, S., and Imaizumi, K. (2012). Obesity-induced endoplasmic reticulum stress causes chronic inflammation in adipose tissue. Scientific Reports 2, 799.

[f] Schaap, F.G., Kremer, A.E., Lamers, W.H., Jansen, P.L. and Gaemers, I.C. (2013). Fibroblast growth factor 21 is induced by endoplasmic reticulum stress. Biochimie 95 (4), 692-699.

SUPPLEMENTARY REFERENCES

[1] Zierler KA, Jaeger D, Pollak NM, Eder S, Rechberger GN, Radner FPW, et al. Functional cardiac lipolysis in mice critically depends on comparative gene identification-58. J. Biol. Chem. 2013;288:9892–904.

[2] Radner FPW, Streith IE, Schoiswohl G, Schweiger M, Kumari M, Eichmann TO, et al. Growth retardation, impaired triacylglycerol catabolism, hepatic steatosis, and lethal skin barrier defect in mice lacking comparative gene identification-58 (CGI-58). J. Biol. Chem. 2010;285:7300–11.

[3] Abel ED, Peroni O, Kim JK, Kim YB, Boss O, Hadro E, et al. Adipose-selective targeting of the GLUT4 gene impairs insulin action in muscle and liver. Nature. 2001;409:729–33.

[4] Sitnick MT, Basantani MK, Cai L, Schoiswohl G, Yazbeck CF, Distefano G, et al. Skeletal muscle triacylglycerol hydrolysis does not influence metabolic complications of obesity. Diabetes. 2013;

[5] Eguchi J, Wang X, Yu S, Kershaw EE, Chiu PC, Dushay J, et al. Transcriptional control of adipose lipid handling by IRF4. Cell Metab. 2011;13:249–59.

[6] Eichmann TO, Kumari M, Haas JT, Farese R V, Zimmermann R, Lass A, et al. Studies on the substrate and stereo/regioselectivity of adipose triglyceride lipase, hormone-sensitive lipase, and diacylglycerol-O-acyltransferases. J. Biol. Chem. 2012;287:41446–57.

[7] Lass A, Zimmermann R, Haemmerle G, Riederer M, Schoiswohl G, Schweiger M, et al. Adipose triglyceride lipase-mediated lipolysis of cellular fat stores is activated by CGI-58 and defective in Chanarin-Dorfman Syndrome. Cell Metab. 2006;3:309–319.

[8] Zimmermann R, Strauss JG, Haemmerle G, Schoiswohl G, Birner-Gruenberger R, Riederer M, et al. Fat mobilization in adipose tissue is promoted by adipose triglyceride lipase. Science (80-.). 2004;306:1383–1386.

[9] Mayer N, Schweiger M, Romauch M, Grabner GF, Eichmann TO, Fuchs E, et al. Development of small-molecule inhibitors targeting adipose triglyceride lipase. Nat. Chem. Biol. 2013;9:785–7.

[10] Folch J, Lees M, Sloane Stanley GH. A simple method for the isolation and purification of total lipides from animal tissues. J. Biol. Chem. 1957;226:497–509.

[11] Zandbergen F, Mandard S, Escher P, Tan NS, Patsouris D, Jatkoe T, et al. The G0/G1 switch gene 2 is a novel PPAR target gene. Biochem. J. 2005;392:313–24.

[12] Lee JH, Giannikopoulos P, Duncan SA, Wang J, Johansen CT, Brown JD, et al. The transcription factor cyclic AMP-responsive element-binding protein H regulates triglyceride metabolism. Nat. Med. 2011;17:812–5.
